# Ezrin and E-cadherin expression profile in cervical cytology: a prognostic marker for tumor progression in cervical cancer

**DOI:** 10.1186/s12885-018-4243-7

**Published:** 2018-03-27

**Authors:** Ana E. Zacapala-Gómez, Napoleón Navarro-Tito, Luz del C. Alarcón-Romero, Carlos Ortuño-Pineda, Berenice Illades-Aguiar, Eduardo Castañeda-Saucedo, Julio Ortiz-Ortiz, Olga L. Garibay-Cerdenares, Marco A. Jiménez-López, Miguel A. Mendoza-Catalán

**Affiliations:** 1 Laboratorio de Biomedicina Molecular, Facultad de Ciencias Químico Biológicas, Universidad Autónoma de Guerrero, Av. Lazaro Cardenas s/n, Ciudad Universitaria, CP, 39090 Chilpancingo, Guerrero Mexico; 2 Laboratorio de Biología Celular del Cáncer, Facultad de Ciencias Químico Biológicas, Universidad Autónoma de Guerrero, Chilpancingo, Guerrero Mexico; 3 Laboratorio de Citopatología e Histoquímica, Facultad de Ciencias Químico Biológicas, Universidad Autónoma de Guerrero, Chilpancingo, Guerrero Mexico; 4 Laboratorio de Ácidos nucleicos y proteínas, Facultad de Ciencias Químico Biológicas, Universidad Autónoma de Guerrero, Chilpancingo, Guerrero Mexico; 5 Instituto Estatal de Cancerología, Acapulco, Guerrero Mexico

**Keywords:** Ezrin, E-cadherin, Cervical cancer, HPV, SIL, Cervical cytology, Immunomarker

## Abstract

**Background:**

Cervical cancer (CC) is the fourth cause of mortality by neoplasia in women worldwide. The use of immunomarkers is an alternative tool to complement currently used algorithms for detection of cancer, and to improve selection of therapeutic schemes. Aberrant expression of Ezrin and E-cadherin play an important role in tumor invasion. In this study we analyzed Ezrin and E-cadherin expression in liquid-based cervical cytology samples, and evaluated their potential use as prognostic immunomarkers.

**Methods:**

Immunocytochemical staining of Ezrin and E-cadherin was performed in cervical samples of 125 patients. The cytological or histological diagnostic was performed by Papanicolaou staining or H&E staining, respectively. HPV genotyping was determined using INNO-LIPA Genotyping Extra kit and the HPV physical status by in situ hybridization. Ezrin expression in HaCaT, HeLa and SiHa cell lines was determined by immunocytochemistry, immunofluorescence and Western blot.

**Results:**

High Ezrin expression was observed in cervical cancer samples (70%), samples with multiple infection by HR-HPV (43%), and samples with integrated viral genome (47%). High Ezrin expression was associated with degree of SIL, viral genotype and physical status. In contrast, low E-cadherin expression was found in cervical cancer samples (95%), samples with multiple infection by HR-HPV/LR-HPV (87%) and integrated viral genome (72%). Low E-cadherin expression was associated with degree of SIL and viral genotype. Interestingly, Ezrin nuclear staining was associated with degree of SIL and viral genotype. High Ezrin expression, high percent of nuclear Ezrin and low E-cadherin expression behaved as risk factors for progression to HSIL and cervical cancer.

**Conclusions:**

Ezrin and E-cadherin expression profile in cervical cytology samples could be a potential prognostic marker, useful for identifying cervical lesions with a high-risk of progression to cervical cancer.

**Electronic supplementary material:**

The online version of this article (10.1186/s12885-018-4243-7) contains supplementary material, which is available to authorized users.

## Background

Cervical cancer (CC) is the fourth cause of death by neoplasms in women worldwide, with a mortality rate of 6.8 per 100,000 women. The highest incidence of CC occurs in less developed regions. In Mexico, CC is the second cause of death by cancer in women, with a mortality rate of 8.1 per 100,000. In southern Mexico, CC associated mortality rate is 14.2 deaths per 100,000 women [1].

Colposcopy and the Papanicolaou staining (Pap test) in liquid-based cytology are the first line screening methods for the early detection of cervical cancer and premalignant lesions [2]. However, the high rate of false positive results of these techniques has led to over intervention, with negative consequences for treated women. Implementation of molecular and cellular biology techniques has improved the specificity and sensitivity of diagnosis, prognosis and treatment of cervical cancer. Nucleic acid amplification and hybridization techniques are used to detect the presence and physical status of viral DNA [3]. On the other hand, immunodetection techniques can be used to evaluate the levels of cellular proteins involved in cell cycle such as p16ink4a and Ki-67 [4], cyclins [5], MCM2 and TOPIIA [6], Temolerase [7], which are markers of HPV infection-associated alterations. Nonetheless, these immunomarkers do not distinguish between transient and persistent (carcinogenic) HPV infections [8].

Proteins of the Ezrin/Radixin/Moesin (ERM) family, participate in the regulation of cell networks associated with tumor progression, through their interaction with membrane proteins, the actin cytoskeleton and signaling molecules, such as CD43, CD44, ICAM-1, ICAM-2 or EGFR [9, 10]. Ezrin is the most studied member of the family, it is expressed in epithelial cells and its overexpression has been reported in breast cancer [11], prostate cancer [12], hepatocellular carcinoma [13], ovarian cancer [14], and endometrial cancer [15]. Ezrin overexpression is associated with poor prognosis and tumor invasiveness. Recently, it has been reported that Ezrin is overexpressed in cervical cancer or intraepithelial neoplasia (CIN) compared to normal cervical tissue [16–19]. Interestingly, Ezrin expression was higher in invasive cancer and metastatic cancer [18]. Ezrin expression is involved in cell migration and invasion in cervical cancer cell lines HeLa and SiHa, it has been suggested that Ezrin could promote cervical cancer progression through regulation of epithelial-mesenchymal transition [20].

Epithelial cell migration requires the dissociation of cell-cell contacts, which are regulated by homotypic interactions between E-cadherin from adjacent cells [9]. Deregulation of E-cadherin expression and its function in invasion and metastasis has been demonstrated in small cell lung cancer [21], breast cancer [22], colon cancer [23] and cervical cancer [24]. In human colon cancer cells transfected to express HPV-16 E6 and E7 oncoproteins, E-cadherin expression is reduced, mainly through E6 protein [25]. Moreover, it has been shown that Ezrin phosphorylation (pY477) could induce the destabilization of adherents junctions and E-cadherin internalization, contributing to cell migration and invasion, suggesting a link between Ezrin and E-cadherin with cancer progression.

Aberrant expression of Ezrin and E-cadherin has been demonstrated in several cervical cancer cell lines and tumor tissue. However, the early detection of cervical cancer (premalignant lesions) is carried out on samples of cervical cytology. In the present study, we evaluated the prognostic value of Ezrin and E-cadherin expression on cervical cytology, as well as its relationship with the genotype and physical status of HPV for the screening of cervical lesions.

## Methods

### Sample collection and diagnosis

One hundred and twenty-five Pap smears were obtained from women who utilized the CC screening service at the Facultad de Ciencias Químico-Biológicas of Universidad Autónoma de Guerrero (UAGro) and the Instituto Estatal de Cancerología of Guerrero state, Mexico. All participants signed an informed consent and a survey was carried out that included sociodemographic and gynecological information. Cervical smears were obtained from transformation zone and exfoliated cells were collected in: 1) DNA extraction solution to perform HPV genotyping; 2) liquid-based cytology to determine the physical status of HR-HPV and proteins expression and; 3) silanized glass slide for cytomorphological examination using the Papanicolaou technique. The diagnosis was done by a certified pathologist or cytopathologist. The Bethesda System was used to define the grade of squamous intraepithelial lesion or cervical cancer. Ethics approval to conduct this study was obtained from the Institutional Ethics Committee at the Universidad Autónoma de Guerrero and the Instituto Estatal de Cancerología.

### HPV detection and genotyping

DNA extraction was performed by SDS-Proteinase K-phenol-chloroform method. Viral genotyping was performed using the INNO-LiPA® Genotyping Extra kit (Innogenetics) according to manufacturer’s instructions. Briefly, the L1 region of HPV was PCR amplified with the SPF10 primers, the biotinylated amplicons were denatured and hybridized with specific and immobile oligonucleotides anchored to a membrane, streptavidin conjugated with alkaline phosphatase was added followed by the chromogen BCIP/NBT to reveal the reaction. The HLA-DPB1 gene was used as a control for DNA amplification, and L1 region of HPV 6 was used as a positive control.

### In situ hybridization

The physical status of High-risk HPV (HR-HPV) was determined by in situ *Hybridization* with a tyramide signal amplification system (GenPonint Dako Cytomation, Carpinteria, CA, US). Cervical smears previously placed in monolayer on silanized slides were fixed with acetone and digested with proteinase K (1: 1000), the two probes of biotilinated viral DNA were added which recognized 13 genotypes of HR-HPV (16, 18, 31, 33, 39, 45, 51, 52, 56, 58, 59 and 68) and 2 LR-HPV genotypes (6 and 11). The slides were subjected to DNA denaturation (10 min at 95 °C) and hybridization for 20 h at 37 °C (Dako Hybridizer, Carpinteria, CA, US). The slides were then placed in an astringent solution, the primary streptavidine peroxidase was added and subsequently biotinyl-tyramide, then the secondary streptavidin and finally the DAB chromogen; cells were counterstained with Mayer’s Hematoxylin. The positive signal for In situ *Hybridization* was visualized as a brown deposit. To determine HPV physical status, a diffuse signal indicated episomal status, a punctiform signal integrated status and a diffuse-punctiform signal a mixed status (DakoCytomation Protocol).

### Immunocytochemistry for Ezrin and E-cadherin expression

Expression of Ezrin and E-cadherin proteins was determined by immunocytochemistry using the streptavidin-biotin peroxidase technique (Bio SB, Mouse/Rabbit ImmunoDetector HRP). For liquid-based citology samples, smears were prepared on silanized glass slides and fixed with 96% and 70% ethanol. For HaCaT (non-tumoral), HeLa (cervical cancer, HPV-18 positive) or SiHa (cervical cancer, HPV-16 positive) cell lines, 5 × 10^4^ cells were plated on glass coverslips in 6-well culture plates and allowed to grow for 24 h, in DMEM medium (Invitrogen, Carlsbad, CA,) supplemented with 10% FBS (Byproductos, Mexico). Cells were fixed with 4% formaldehyde; endogenous peroxidase was blocked with 3% hydrogen peroxide for 20 min and non-specific protein binding was performed with 1% BSA in PBS for 40 min. The slides were incubated with primary anti-Ezrin antibody (1:100 dilution; clone 3C12, Santa Cruz Biotechnology ScC-58,758) or anti-E-cadherin antibody (1:50 dilution; Santa Cruz Biotechnology SC-7870) in a humidity chamber during 1 h at room temperature. Biotin-bound secondary antibody was added for 30 min at room temperature followed by incubation with diaminobenzidine (DAB) for 1-2 min. Cells were counterstained with Mayer’s Hematoxylin for 10 min and the samples were dehydrated in descending degrees of ethanol and finally mounted with fast mounting medium Entellan (MERCK) and observed under a brightfield microscope (Olympus BX-43). As a negative control, we used samples processed under the same conditions as those already described, but in the absence of primary antibodies. Protein expression was visualized as brown signal and the intensity of staining was scored as low, moderate or high. To analyze the nuclear staining index, the number of cells with positive nuclei to Ezrin was divided among the number total cells present in the slide and multiplied by 100; this nuclear staining index was divided into tertiles (< 50%, 50-89% and > 90%). All the preparations were independently evaluated by three analysts, without knowing the cytological diagnosis, to establish a consensus about protein expression for each sample.

### Immunofluorescence

5 × 10^4^ cells (HaCaT, HeLa or SiHa) were plated on glass coverslips in 6-well culture plates and allowed to grow for 24 h in DMEM HamF/12 medium (Invitrogen, Carlsbad, CA,) supplemented with 10% FBS (Byproductos, Mexico) at 37 °C in a 5% CO_2_ atmosphere. Cells were fixed with ice-cold methanol (− 20 °C) for 5 min, and permeabilized with PBS + 0.2% Triton X-100. The non-specific bindings were blocked with PBS + 1% BSA for 40 min and the samples were incubated with primary anti-Ezrin antibody (1:100 dilution; clone 3C12, Santa Cruz Biotechnology ScC-58,758) in a humidified chamber for 1 h at room temperature. Subsequently, FITC-conjugated secondary anti-mouse antibody (1:50 dilution) was added for 30 min in a humidified/dark chamber at room temperature and samples were mounted using ProLong Gold Antifade Mountant with DAPI (Invitrogen, P-36931). The images were acquired and processed on an EVOs FL Auto microscope (Thermo Fisher Scientific).

### Protein extraction and western blot

HaCaT, HeLa or SiHa cells were seeded on 100 mm plates and allowed to grow until 80% confluency. Cells were washed with PBS and lysed with RIPA buffer (50 mM Tris-HCl pH 7.6, 160 mM NaCl, 0.5 mM EDTA/EGTA, 1% Triton X-100, 10% glycerol, 1 mM PMSF and 1 μg/ml leupeptin). Protein extracts were separated by SDS-PAGE in 8% acrylamide gels, transferred to PVDF membranes and incubated overnight at 4 °C with antibodies anti-Ezrin (1:1000 dilution; clone 3C12, Santa Cruz Biotechnology) or anti-Tubulin (Merck Millipore) as loading control, overnight at 4 °C. The membrane was incubated with a secondary anti-mouse antibody (1:3000 dilution, Merck Millipore) at room temperature and revealed with chemiluminescent substrate (Luminata, Millipore).

### Statistical analysis

Data ware expressed as absolute and relative frequencies. The association between variables was calculated using chi-square test (χ2). A logistic regression analysis was performed to calculate Odds Ratios (OR) and confidence intervals (CI) at 95% comparing the Ezrin and E-cadherin expression in non-SIL and LSIL group versus HSIL and CC group. To evaluate differences in Ezrin expression level between HaCaT and HeLa or SiHa cells, *t-student* test was used. *P* value < 0.05 was considered statistically significant.

## Results

The study population consisted of 125 patients from southern Mexico: 20 Non-SIL and HPV negative, 24 Non-SIL and positive for HPV, 41 with L-SIL, 20 with H-SIL and 20 with CC. The mean age of the patients was 41 years (range 21-76). The clinical and pathological characteristics are summarized in Table 1.

**Table 1:** Clinical and pathological characteristics of study population

Characteristics	Cytological diagnosis					
	Non-SIL, HPV (−)	Non-SIL, HPV (+)	LSIL	HSIL	Cervical cancer	^*^p value
	% (*n*)	% (*n*)	% (*n*)	% (*n*)	% (*n*)	
Age (years)						
21 - 30	35 (7)	25 (6)	27 (11)	25 (5)	0 (0)	
31 - 40	35 (7)	33 (8)	29 (12)	30 (6)	20 (4)	0.050
41 - 50	25 (5)	33 (8)	22 (9)	15 (3)	30 (6)	
> 50	5 (1)	9 (2)	22 (9)	30 (6)	50 (10)	
Total	20	24	41	20	20	
Beginning sexual activity (years)^α^						
12 - 16	35 (7)	13 (3)	19 (8)	35 (7)	37 (7)	
17 - 19	35 (7)	29 (7)	37 (15)	45 (9)	16 (3)	0.147
> 20	30 (6)	58 (14)	44 (18)	20 (4)	47 (9)	
Total	20	24	41	20	19	
Menarche (years) ^α^						
< 12	35 (7)	33 (8)	15 (6)	10 (2)	5 (1)	0.155
12 - 13	35 (7)	50 (12)	48 (19)	50 (10)	58 (11)	
> 13	30 (6)	17 (4)	37 (15)	40 (8)	37 (7)	
Total	20	24	40	20	19	
Active Smoker^α^						
Yes	85 (17)	75 (18)	93 (38)	85 (17)	88 (15)	0.399
No	15 (3)	25 (6)	7 (3)	15 (3)	12 (2)	
Total	20	24	41	20	17	
Alcohol consumption^α^						
Yes	0 (0)	17 (4)	10 (4)	0 (0)	0 (0)	0.069
No	100 (20)	83 (20)	90 (37)	100 (20)	100 (17)	
Total	20	24	41	20	17	
Menopause^α^						
No-menopausal stage	74 (14)	83 (19)	74 (28)	74 (14)	35 (6)	
< 50 or less	21 (4)	13 (3)	16 (6)	21 (4)	59 (10)	0.043
> 50	5 (1)	4 (1)	11 (4)	5 (1)	6 (1)	
Total	19	23	38	19	17	

^α^Missing information; some patients did not answer the question. ^*^Chi-square test

### Ezrin and E-cadherin expression is related to cytological diagnosis and HPV genotype

**Fig. 1:**
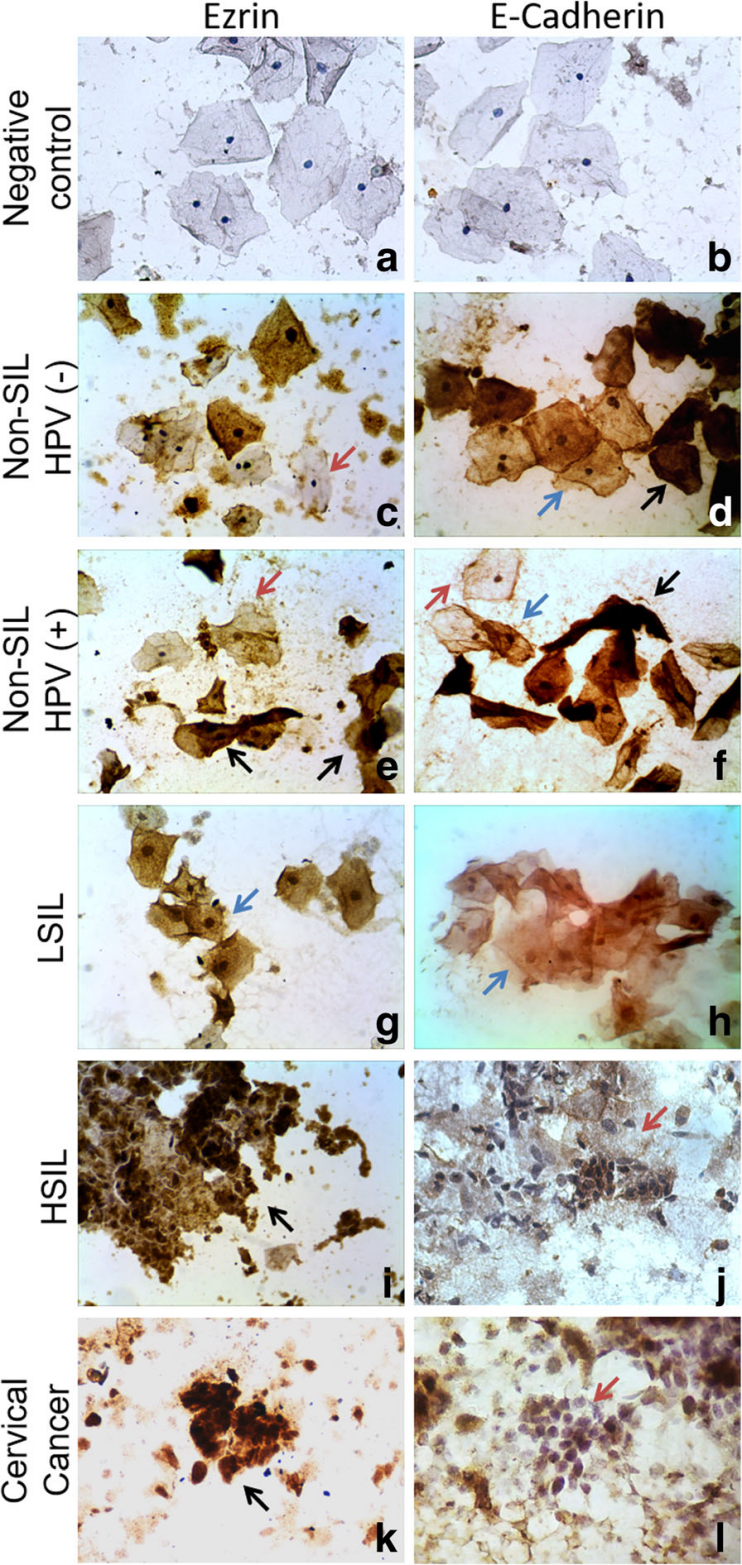
Ezrin and E-cadherin expression in cervical cytology samples. Immunocytochemical staining using streptavidin-biotin peroxidase technique. **a** and **b** (Negative control): Non-SIL samples without primary antibody, 60X. **c** to **l**, 40X. Red arrow: Negative/low expression; Blue arrow: moderate expression; Black arrow: high expression of Ezrin or E-cadherin

Regardless of the diagnosis, Ezrin and E-cadherin expression was higher in basal and parabasal cells, compared to intermediate and superficial cells, which showed low expression of both proteins. Ezrin expression was low in non-SIL samples and high in HSIL or CC samples. In contrast, E-cadherin staining showed moderate/high intensity in non-SIL and LSIL, and low intensity in HSIL and CC (Fig. 1).

Ezrin and E-cadherin expression was significantly associated to the degree of SIL (< 0.001). 70% of cervical cancer samples show high expression of Ezrin, while 65% of non-SIL HPV (−) samples show low/negative expression of Ezrin. In the presence of HPV infection, even in absence of SIL the expression of Ezrin changes from low to moderate (58% of cases). In contrast, 95% and 75% of cervical cancer and HSIL samples, respectively, showed low or negative E-cadherin expression, whereas moderate/high expression of E-cadherin was observed in LSIL or non-SIL samples (Table 2). Moreover, we found that Ezrin and E-cadherin expression are correlated with p16ink4a and Ki-67 expression, which are validated markers at the detection of cervical lesions; in the cases that showed high expression of p16ink4a and Ki-67, the Ezrin expression was majorly high and E-cadherin expression was predominantly low (Additional file 1: Table S1). On the other hand, Ezrin and E-cadherin expression was significantly correlated to HPV infection (*p* < 0.001); high expression of Ezrin and low/negative expression of E-cadherin was observed in samples with multiple infections: HR-HPV and LR-HPV or several HR-HPV (Table 2). The changes in Ezrin and E-cadherin expression were related to presence of HPV-16 mainly; 95% of samples positive for HPV-16 showed high Ezrin expression and 74% showed low E-cadherin expression, and no differences were observed in expression of these proteins in relation to HPV-18 or other HPV-AR genotypes (Additional file 2: Table S2). A statistically significant correlation was observed between the HR-HPV integration and Ezrin expression; furthermore, Ezrin expression was high in 47% and 29% of samples with integrated or mixed HR-HPV genome, respectively. In contrast, E-cadherin expression was independent of physical status of HR-HPV (Table 2). We observed cases of HR-HPV integration in No SIL and LSIL group that were correlated with an increase in Ezrin expression (Additional file 3: Table S3).

**Table 2:** Ezrin and E-cadherin expression and their relation to cytological diagnosis, HPV infection and viral integration

	Total	Ezrin expression			^*^p value	E-cadherin expression			
		Neg/Low	Moderate	High		Neg/Low	Moderate	High	^*^p value
	*n*	% (*n*)	% (*n*)	% (*n*)		% (*n*)	% (*n*)	% (*n*)	
Diagnosis									
Non-SIL, HPV (−)	20	65 (13)	35 (7)	0 (0)	< 0.001	35 (7)	35 (7)	30 (6)	< 0.001
Non-SIL, HPV (+)	24	33 (8)	58 (14)	8 (2)		25 (6)	54 (13)	21 (5)	
LSIL	41	44 (18)	39 (16)	17 (7)		32 (13)	68 (28)	0 (0)	
HSIL	20	25 (5)	30 (6)	45 (9)		75 (15)	15 (3)	10 (2)	
Cervical cancer	20	0 (0)	30 (6)	70 (14)		95 (19)	5 (1)	0 (0)	
HPV infection									
HR-HPV	45	38 (17)	40 (18)	22 (10)	< 0.001	58 (26)	38 (17)	4 (2)	< 0.001
MI HR-HPV	30	13 (4)	43 (13)	43 (13)		60 (18)	23 (7)	17 (5)	
MI HR + LR-HPV	8	0 (0)	25 (2)	75 (6)		87 (7)	12 (1)	0 (0)	
LR-HPV	20	50 (10)	45 (9)	5 (1)		0 (0)	100 (20)	0 (0)	
Negative	22	59 (13)	32 (7)	9 (2)		41 (9)	32 (7)	27 (6)	
HPV Physical status									
Episomal	5	80 (4)	20 (1)	0 (0)	0.021	60 (3)	40 (2)	0 (0)	0.366
Integrated	32	25 (8)	28 (9)	47 (15)		72 (23)	19 (6)	9 (3)	
Mixed	41	22 (9)	49 (20)	29 (12)		51 (21)	39 (16)	10 (4)	

*MI* Multiple infection, two or more viral types. ^*^Chi-square test

### Ezrin nuclear localization is associated to HSIL and cervical cancer

**Fig. 2:**
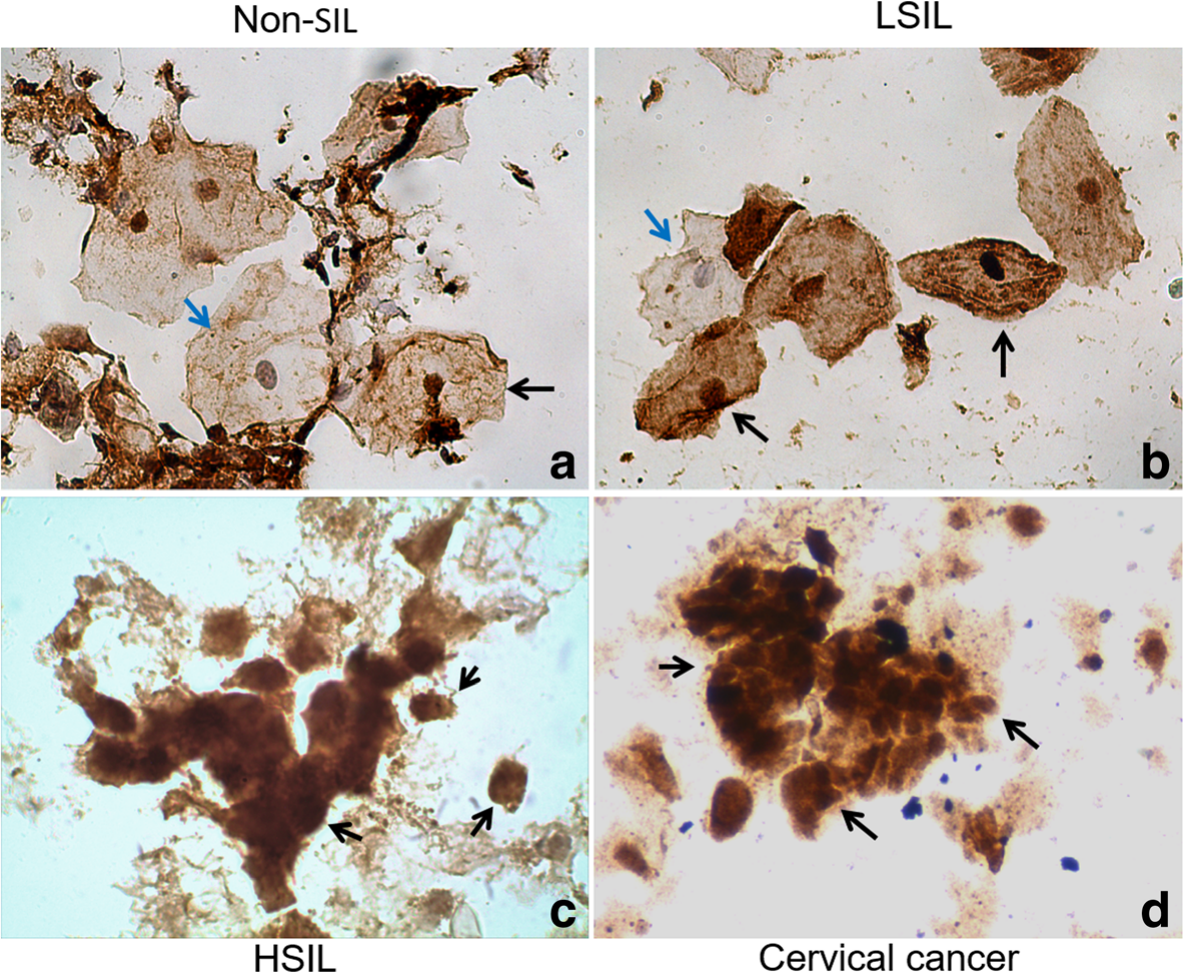
Subcellular localization of Ezrin in squamous intraepithelial lesions and cervical cancer. Immunocytochemistry for Ezrin protein, 60×. **a** Non-SIL, **b** LSIL, **c** HSIL, **d** cervical cancer. Black arrow: cell with positive nuclei for Ezrin. Blue arrow: cell negative to Ezrin nuclear

Interestingly, nuclear staining for Ezrin was observed more frequently in HSIL and CC samples (Fig. 2). A statistical relation between nuclear Ezrin and cytological diagnosis was found (*p* < 0.001). 85% and 60% of CC and HSIL samples, respectively, showed > 90% of cells with nuclear Ezrin, whereas only 30-33% of non-SIL and 20% of LSIL samples were grouped in this category (Table 3). Moreover, Ezrin nuclear localization showed a statistically significant correlation with the HPV genotype (*p* = 0.001); 49% of samples positive for HR-HPV infection showed > 90% of cells with nuclear Ezrin, while 60% of the LR-HPV samples showed < 50% of cells with Ezrin-positive nuclei. No association was found between HR-HPV physical status and nuclear Ezrin (Table 3).

**Table 3:** Nuclear staining of Ezrin and its relation to cytological diagnosis, HPV infection and viral integration

		Nuclei positive to Ezrin			^*^ *p value*
	Total	< 50%	50-89%	> 90%	
	*n*	% (*n*)	% (*n*)	% (*n*)	
Diagnosis					< 0.001
Non-SIL, HPV (−)	20	20 (4)	50 (10)	30 (6)	
Non-SIL, HPV (+)	24	33 (8)	33 (8)	33 (8)	
LSIL	41	29 (12)	51 (21)	20 (8)	
HSIL	20	10 (2)	30 (6)	60 (12)	
Cervical cancer	20	10 (2)	5 (1)	85 (17)	
Viral genotype					
HR-HPV	45	18 (8)	33 (15)	49 (22)	0.001
MI HR-HPV	30	13 (4)	40 (12)	47 (14)	
MI HR + LR-HPV	8	0 (0)	25 (2)	75 (6)	
LR-HPV	20	60 (12)	35 (7)	5 (1)	
Negative	22	18 (4)	45 (10)	36 (8)	
HPV Physical status					
Episomal	5	20 (1)	60 (3)	20 (1)	0.115
Integrated	32	16 (5)	19 (6)	66 (21)	
Mixed	41	15 (6)	44 (18)	41 (17)	

*MI* Multiple infection, two or more viral types. ^*^Chi-square test

### High Ezrin and low E-cadherin expression are associated with diagnosis of HSIL and cervical cancer

To evaluate the risk conferred by high Ezrin and low E-cadherin expression for development of HSIL and CC, we calculated ORs using a bivariate analysis comparing non-SIL/SIL group versus HSIL/CC group. We found that high Ezrin and low E-cadherin expression implicate 4.61 and 6.14 times more risk, respectively, for developing HSIL or CC (Table 4), suggesting that combined determination of Ezrin and E-cadherin expression could be useful for assessing the prognosis of patients. Another interesting fact that could be considered as a prognostic marker is the high percentage of nuclear staining to Ezrin, which implicates 3.68 times more risk of developing a HSIL or CC (Table 4).

**Table 4:** Ezrin and E-cadherin expression and their association with development of HSIL and CC

	Non-SIL / LSIL	HSIL / CC	Non-SIL / LSIL^b^	HSIL / CC	*p*
	% (*n*)	% (*n*)	OR^a^	OR^a^ (95% CI)	
Ezrin					
Neg/Low^b^	46 (39)	12 (5)	1.0	4.61 (2.36-8.96)	< 0.001
Moderate	43 (37)	30 (12)			
High	11 (9)	58 (23)			
E-cadherin					
Neg/Low	31 (26)	85 (34)	1.0	6.14 (2.59-14.54)	< 0.001
Moderate	56 (48)	10 (4)			
High^b^	13 (11)	5 (2)			
Ezrin-positive nuclei					
< 50%^b^	28 (24)	10 (4)	1.0	3.68 (1.89-7.17)	< 0.001
50-89%	46 (39)	17 (7)			
≥ 90%	26 (22)	73 (29)			

^a^*OR* Odds ratio adjusted for age, ^b^Indicate reference category, *CC* cervical cancer, *CI* confidence interval

**Fig. 3:**
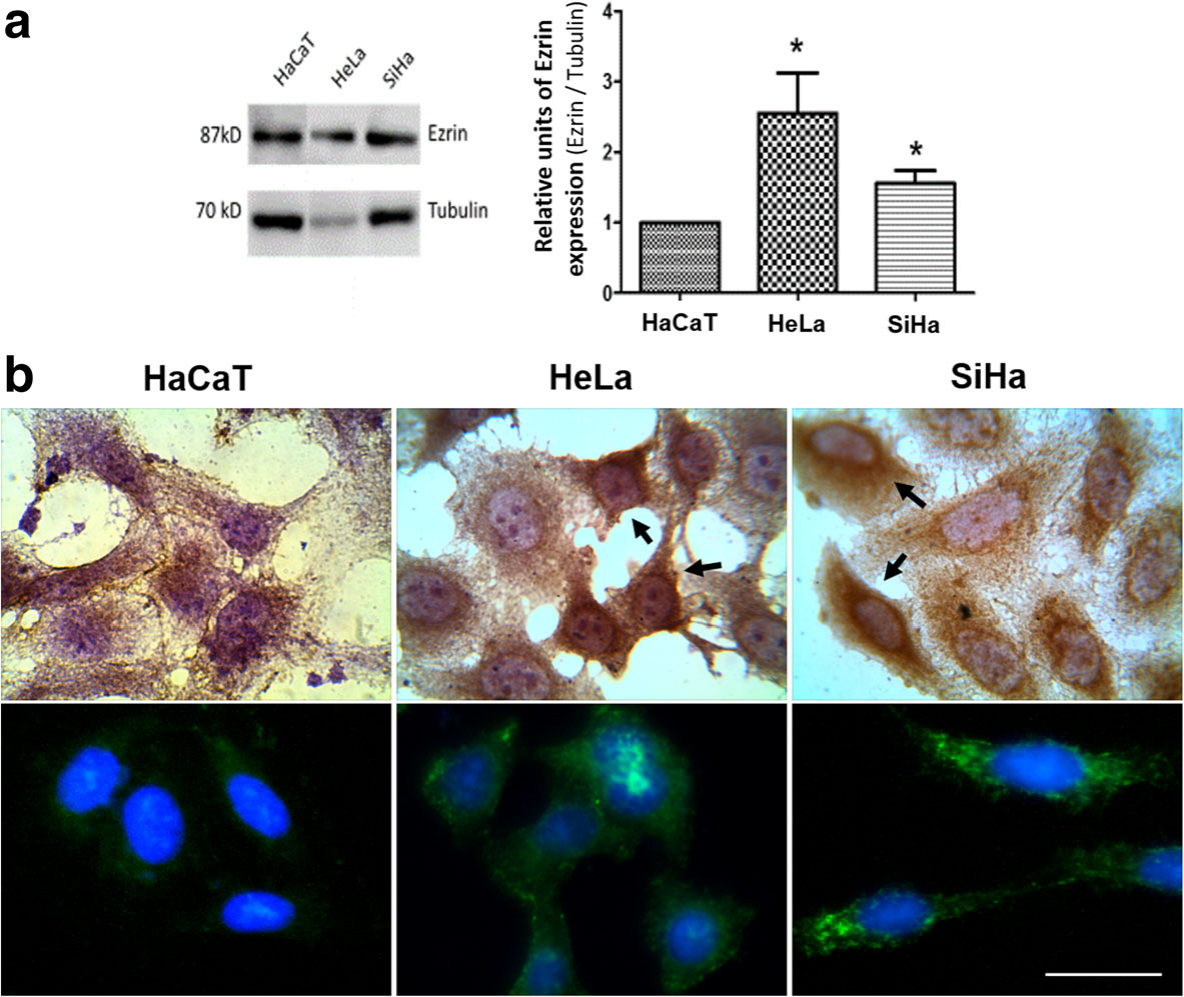
Ezrin expression in cervical cell lines in vitro. **a** Western blot for Ezrin expression (left) and densitometry corresponding to three independent replicates (right), *t-student test, *p* value< 0.05. **b** Top panel: Immunocytochemistry (100×), black arrow indicates perinuclear staining; bottom panel: immunofluorescence (100X). Scale bar represents 25 μm

In cervical cancer cells in vitro, Ezrin expression was higher in HeLa and SiHa cells compared to non-tumor cells HaCaT (Fig. 3a). Moreover, in HaCaT cells Ezrin immunoreactivity was exclusively cytoplasmic, whereas in HeLa and SiHa cells a perinuclear staining of Ezrin was observed, mainly in those cells where Ezrin expression was higher (Fig. 3b).

## Discussion

Many studies have proposed biomarkers to differentiate normal tissue and tumor tissue, or non-SIL and SIL. However, no immunomarkers have been reported to distinguish between cervical lesions that will regress and lesions with high potential of progression to invasive cervical cancer. The objective of this study was to analyze the usefulness of Ezrin and E-cadherin expression as prognostic biomarkers for development of HSIL and cervical cancer using liquid-based cytology samples. We showed that high Ezrin expression and low E-cadherin expression are associated with the risk of progression to HSIL and cervical cancer. In addition Ezrin expression was associated with HPV integration.

Here, we observed a high Ezrin expression in HSIL and cervical cancer samples compared to non-SIL and HPV negative samples. These results are in agreement with the data reported by Tan et al.*,* (2011) and Kong et al.*,* (2013), who using immunohistochemistry of paraffin-embedded biopsies showed that more than 80% of cervical cancer samples presented high Ezrin expression compared to non-neoplastic tissue [17, 26]. Our results demonstrate that Ezrin expression can be detected using cervical smears in liquid cytology, a non-invasive method. In addition, we found an association between the HPV genotype and Ezrin expression. The highest Ezrin levels were observed in samples with HR-HPV multiple infections, and mainly in the samples positive for HPV-16. Other studies have reported a relation of Ezrin expression with HPV infection. Shen et al.*,* (2003) reported that E6/E7 expression of HPV-18 in esophageal epithelial cells induced an increase Ezrin expression [27]. Auvinen et al.*,* (2013), reported an increase of Ezrin expression in HPV-associated cervical lesions [16]. In contrast, Kong et al.*,* (2013) found no statistically significant correlation between HR-HPV infection and Ezrin overexpression; however the authors support the idea that there is a positive correlation of HR-HPV infection and Ezrin overexpression in cervical cancer, because more than 80% of HPV-infected cervical cancer samples showed Ezrin overexpression [17]. An important difference could be that in the study by Kong et al.*,* (2013) only Ezrin overexpression and the presence or absence of HPV were compared, whereas in our study we compared Ezrin expression and HPV infection considering the HPV genotype and multiple infections.

It has been demonstrated that Ezrin expression is higher in invasive or metastatic tumors [18, 26], and Ezrin expression is necessary for the invasive ability of cervical tumor cells through induction of epithelial-mesenchymal transition (EMT) [20]. These data are related with the overexpression of Six1, which is a transcription factor for Ezrin, reported in cervical cancer tissue [26], and has been reported that Six1 overexpression promoted EMT at early stages of HPV16-mediated transformation of human keratinocytes [28]. In a study by Sun et al.*,* (2016), they reported that Six1 overexpression increased the sensitivity of tumor cells to TGFβ stimulation inducing EMT in vitro, reducing the E-cadherin expression and increasing the N-cadherin expression [29]. On the other hand, a gradually decreasing E-cadherin expression and gradually increasing P-cadherin expression has been reported in cervical intraepithelial neoplasia (CIN) until squamous cell carcinoma [24]. Similarly, in human colon cancer cells expressing E6 and E7 proteins of HPV16, it was observed that these oncoproteins reduce E-cadherin expression, observing a greater effect with E6 protein [25]. These data are consistent with the observations in our study, because we observed decreased E-cadherin expression in HSIL and CC samples and these changes were related to viral genotype, observing a minor expression when HR-HPV was present. One limitation of our study is the small number of samples, and no data are available on the evolution of patients diagnosed with cervical cancer to evaluate the relationship of Ezrin and E-cadherin expression to invasive tumor capacity and survival of patients.

On the other hand, an interesting observation in our study was the positive nuclear staining for Ezrin in cervical cytology; a high percent of HSIL and CC samples presented more than 90% of cells with Ezrin-positive nuclei, which was associated with risk of progression to HSIL and CC. To our knowledge, we reported for the first time this observation in cervical cells. Halon et al.*,* (2013) reported nuclear localization of Ezrin in breast cancer tissue and it was associated with the presence of nodal metastases and the tumor aggressivity [30]. Kong et al.*,* (2013) observed perinuclear staining of Ezrin in cervical cancer cells, suggesting this Ezrin distribution pattern could be useful as a prognostic marker [17]. In this study, we observed that Ezrin staining was exclusively cytoplasmic in non-tumor cells HaCaT, but was cytoplasmic and perinuclear in cervical cancer cells HeLa and SiHa. According to the above mentioned, these data suggest the potential usefulness of Ezrin immunoreactivity (expression level and subcellular localization) as prognostic marker in cervical cytology.

In our study, we found that an increase of Ezrin expression, high percent of Ezrin nuclear and a decrease of E-cadherin expression are associated with progression to HSIL and CC. Considering that Ezrin overexpression induces the invasive potential of cervical tumors and it is associated with EMT, which is characterized by loss of E-cadherin expression, we propose using the Ezrin and E-cadherin immunostaining profile in liquid-based cytology, a noninvasive test, as predictor of prognosis in patients with squamous intraepithelial lesions.

## Conclusions

In conclusion, detection of Ezrin and E-cadherin expression in cervical smears, could be a potential prognostic marker for identifying cervical lesions with high-risk of progression to invasive cervical cancer, and may help on the selection of an appropriate therapy or avoid unnecessary treatment; a larger number of samples and a follow-up study will help to confirm this proposal.

## Additional files

Additional file 1: **Table S1.** Correlation of Ezrin and E-cadherin expression with validated biomarkers. (TIFF 2264 kb)

Additional file 2: **Table S2.** Association between viral genotype and Ezrin and E-cadherin expression. (TIFF 2623 kb)

Additional file 3: **Table S3.** Physical status of HR-HPV by diagnosis and its correlation with Ezrin and E-cadherin expression. (TIFF 1493 kb)
