# Supplementary figures and images for: Ezrin and E-cadherin expression profile in cervical cytology: a prognostic marker for tumor progression in cervical cancer
Source: BMC Cancer. 2018 Mar 27;18:349. doi: 10.1186/s12885-018-4243-7 (PMC5872531; doi:10.1186/s12885-018-4243-7)

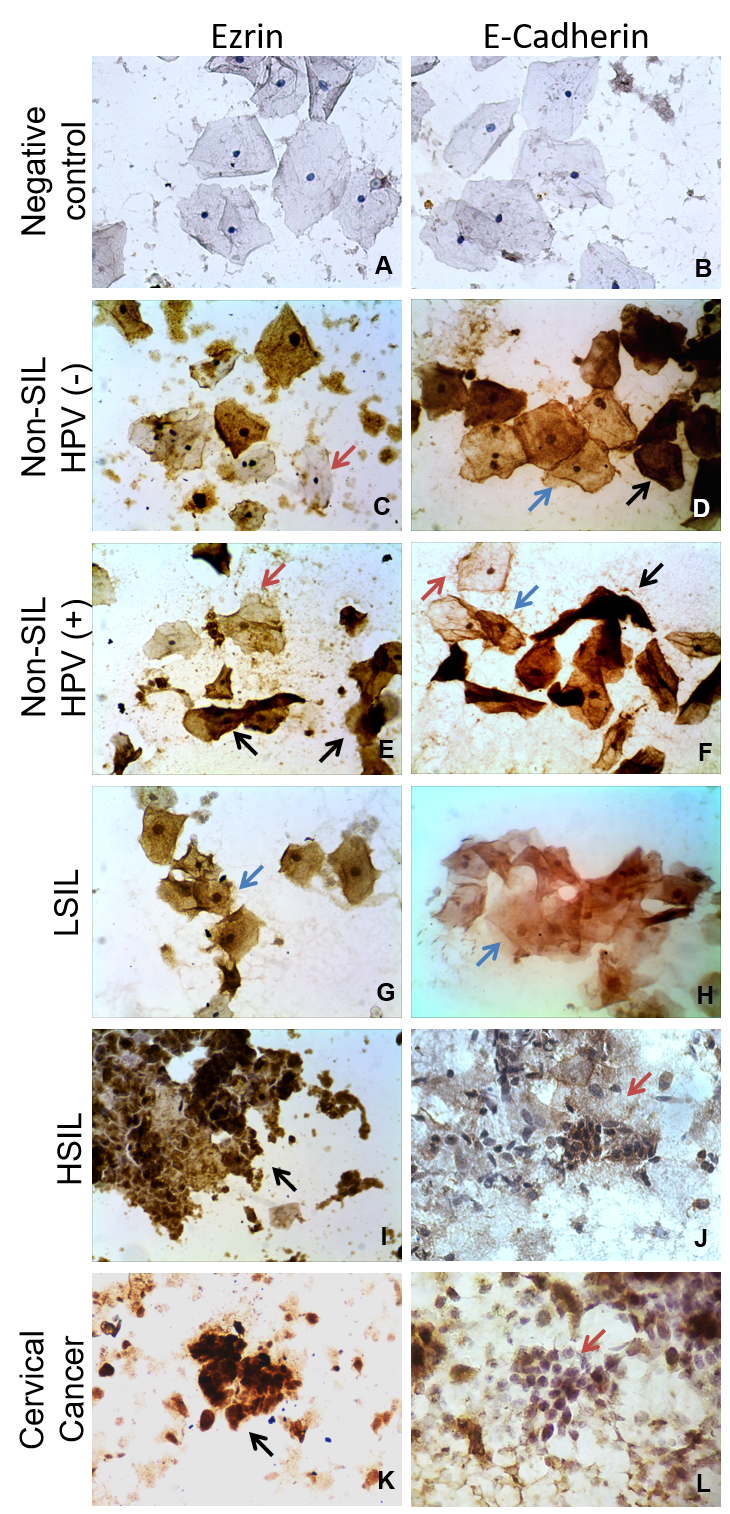

Supplement: Supplementary file 1 — Table S1. Correlation of Ezrin and E-cadherin expression with validated biomarkers. (TIFF 2264 kb) [file 12885_2018_4243_MOESM1_ESM.tif]

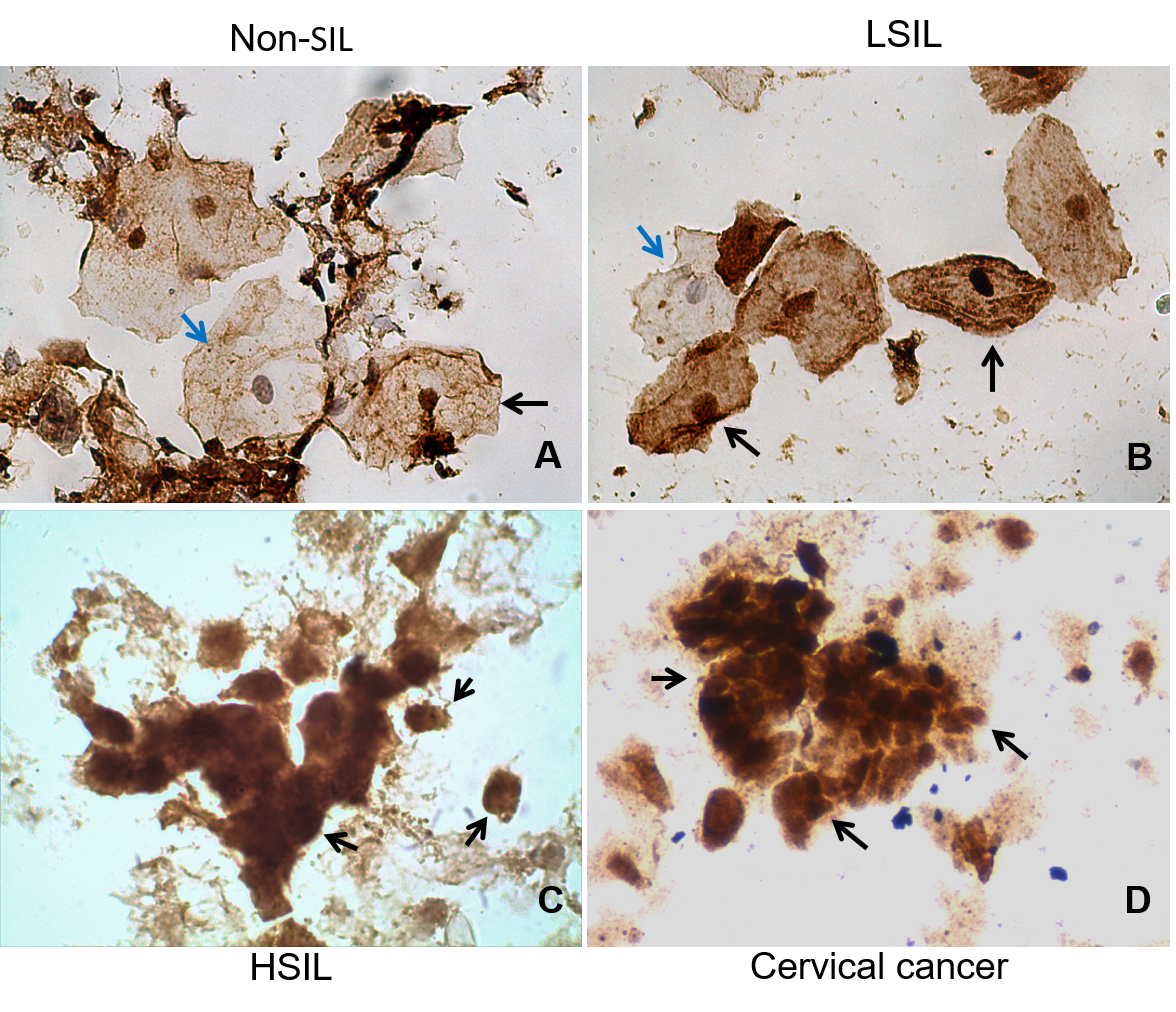

Supplement: Supplementary file 2 — Table S2. Association between viral genotype and Ezrin and E-cadherin expression. (TIFF 2623 kb) [file 12885_2018_4243_MOESM2_ESM.tif]

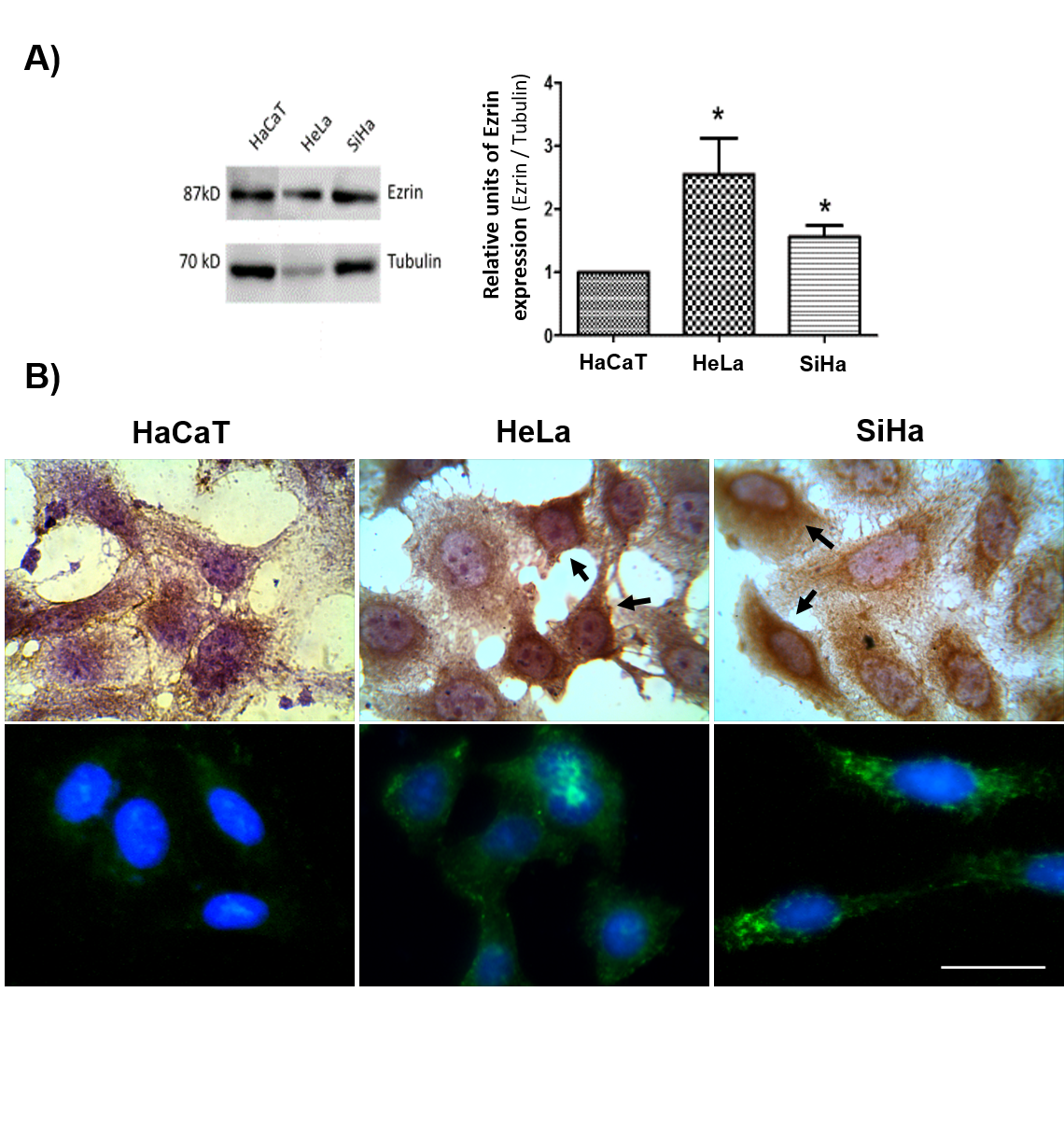

Supplement: Supplementary file 3 — Table S3. Physical status of HR-HPV by diagnosis and its correlation with Ezrin and E-cadherin expression. (TIFF 1493 kb) [file 12885_2018_4243_MOESM3_ESM.tif]
